# ACHIEVING SUSTAINABILITY IN HOUSING WITH SERVICES: INSIGHTS FROM R3 PROGRAM

**DOI:** 10.1093/geroni/igac059.824

**Published:** 2022-12-20

**Authors:** Pamela Nadash, Edward Miller, Elizabeth Simpson, Molly Wylie, Natalie Shellito, Yan Lin, Taylor Jansen, Marc Cohen

**Affiliations:** University of Massachusetts Boston, Boston, Massachusetts, United States; University of Massachusetts Boston, Boston, Massachusetts, United States; University of Massachusetts Boston, Boston, Massachusetts, United States; University of Massachusetts Boston, Boston, Massachusetts, United States; University of Massachusetts Boston, Boston, Massachusetts, United States; University of Massachusetts Boston, Boston, Massachusetts, United States; University of Massachusetts Boston, Boston, Massachusetts, United States; University of Massachusetts Boston, Boston, Massachusetts, United States

## Abstract

There has been a long-standing interest in developing housing models that integrate the supportive services that older people need to remain at home as long as possible. This approach has boomed for better-off people who can afford the cost of private-pay independent living and assisted living environments. For those with fewer resources, however, options are limited, given that they are largely dependent on public financing. This study focuses on how to develop sustainable models of housing with services for low-income older people. Using data from 31 key informant interviews and three focus groups, it reports and analyzes expert perspectives on how programs such as R3 can achieve financial sustainability. Four major themes emerged: (1) funding as the key to sustaining housing with services; (2) funding housing with services through participating health plans; (3) other potential funding sources for housing with services; and (4) gaining buy-in for housing with services.

